# Comorbidity of overweight and obesity in a nationally representative sample of German adults aged 18-79 years

**DOI:** 10.1186/1471-2458-12-658

**Published:** 2012-08-15

**Authors:** Anja Schienkiewitz, Gert B M Mensink, Christa Scheidt-Nave

**Affiliations:** 1 Department of Epidemiology and Health Monitoring, Robert Koch-Institut, General-Pape-Str. 62-66, 12101, Berlin, Germany

## Abstract

**Background:**

Overweight has increased in many countries over the past 20 years and excessive body weight is an established risk factor for adverse health outcomes and chronic diseases. This study aimed to determine comorbidity associated with overweight and obesity in a nationally representative sample of German adults.

**Methods:**

In the German National Health Interview and Examination Survey 1998 standardized measures of body weight, height and waist circumference (WC) were obtained for 7,124 men and women 18 to 79 years of age. Information on pre-existing health conditions, health-related behaviors, and sociodemographic characteristics was collected using physician-administered computer-assisted interviews and self-administered questionnaires. World Health Organization (WHO) cut-off criteria were applied to define overweight (BMI 25.0-29.9 kg/m^2^) and obesity (BMI ≥30.0 kg/m^2^) and abdominal obesity (men: WC ≥102 cm; women: WC ≥88 cm).

**Results:**

The crude prevalence of persons with cardiometabolic risk factors, diabetes mellitus, cardiovascular disease (CVD), gall bladder disease, and osteoarthritis showed a significant stepwise increase from the lowest to the highest BMI category in both sexes. In multiple logistic regression models adjusting for age, social status, and smoking, significant associations with overweight and obesity persisted for cardiometabolic risk factors and osteoarthritis. For example, obese persons had a three- to fourfold higher chance of having any cardiometabolic risk factor compared to normal weight persons (odds ratio (OR) = 4.07, 95% CI: 3.16-5.25 for men; OR = 3.40 (2.60-4.46) for women). Only in women, overweight and obesity as well as abdominal obesity, independent of BMI category, were significantly and consistently associated with diabetes (overweight: OR = 1.85 (1.03-3.30); obesity: OR = 2.94 (1.63-5.31); abdominal obesity: OR = 1.44 (1.08-1.92) and gall bladder disease (overweight: OR = 1.65 (1.22-2.25); obesity: OR = 3.06 (2.26-4.14); abdominal obesity: OR = 1.73 (1.25-2.39)).

**Conclusion:**

Current estimates of disease burden underline the public health importance and clinical relevance related to overweight and obesity and needs to take into account comorbidity aspects.

## Background

Worldwide, the prevalence of persons with overweight or obesity has increased in many countries over the past 20 years [1-4]. Excessive body weight is an established risk factor for adverse health outcomes and chronic diseases [5]. Obesity is not only associated with high blood pressure [6,7], and type 2 diabetes [8,9], but also related to cardiovascular diseases (CVD) independent of blood pressure and lipid levels [10,11]. Results from the Health Professionals Follow-up Study and Framingham Heart Study identified obesity as a risk factor for gout [12,13]. High BMI levels are associated with an increased risk of coronary heart disease [7,14,15] and ischemic stroke [16]. In addition, increased BMI is an established risk factor for several types of cancer [17,18]. A prospective evaluation of the Nurses Health and Health Professionals Follow-up Study indicated a positive association between abdominal adiposity and gallstone diseases in women and men [19,20]. The relationship between obesity and thyroid diseases is more complex as hypothyroidism is associated with weight gain [21]. The prospective Whitehall-II-Study reported results that common mental disorders increase the risk for obesity, but if participants with common mental disorders at baseline were removed from the analysis, no association between obesity and mental disorders could be found [22]. Furthermore, obesity is directly related to osteoarthritis [23,24]. Obesity may also be an important risk factor for allergic disease (e.g. allergic rhinoconjunctivitis, allergic contact exzema, neurodermatitis, food allergy, urticaria), but the relationship remains less clear [25]. Several studies have reported a positive association [26-28], whereas others did not find a relationship between obesity and atopy, hay fever, or serological markers of atopy [29,30].

The above mentioned references indicate that several articles focus on single health outcomes. Only a few studies have used nationally representative survey data to systematically analyze the comorbidities of overweight and obesity [31-33]. In the present study, we used data from the 1998 German National Health Interview and Examination Survey (GNHIES) to examine sex and age specific associations between a large list of self-reported chronic health conditions and overweight and obesity as defined by measures of BMI and waist circumference (WC).

## Methods

### Study design and study population

The German Health Interview and Examination Survey (GNHIES) was conducted from October 1997 to March 1999 as a population-wide, nationally representative cross-sectional survey including 7,124 participants (3,450 men, 3674 women) aged 18 to 79 years. Sampling procedures have previously been described in detail [34]. In brief, the population sample was derived in a two-stage sampling procedure. First, a representative sample of communities with respect to their size and federal state was selected. Second, a random selection of non institutionalized adults from local population registries stratified by sex and 5-year-age groups was performed. The overall response rate was 61.4% [35]. Approval for the survey was given from the Federal Office for the Protection of Data, Germany. Written informed consent was obtained from all participants prior to the interview and examination.

For the present analysis, we excluded a total of 86 participants with missing information or invalid values on anthropometric measurements (weight, height, waist circumference) as well as 25 persons, who did not complete the computer assisted personal interview. Multivariate analyses are based on a total of 6,790 participants (3,320 men, 3,470 women) with complete data. The number of participants in these analyses is slightly reduced due to the exclusion of those who had missing values on social status (n = 217) and smoking (n = 156).

### Anthropometric variables

Standardized measurements of body weight, body height and waist circumference (WC) were conducted with participants wearing only light clothing and no shoes. Body weight was measured with a calibrated electronic scale (type: SECA) to the nearest 0.1 kg and body height was measured with a leveling board on the electronic scale to the nearest 0.1 cm. WC was measured using a flexible, non-stretchable measuring tape to an accuracy of 0.1 cm. Body mass index (BMI) was calculated as body weight (in kg) divided by body height squared (in m). We applied World Health Organization (WHO) recommended criteria to define normal weight (BMI < 25 kg/m^2^), overweight (BMI = 25.0-29.9 kg/m^2^) and obesity (BMI ≥ 30.0 kg/m^2^) and abdominal obesity based on WC (men: ≥102 cm; women: ≥88 cm) [36].

### Assessment of comorbidity

Using a standardized computer-assisted personal interview (CAPI), specifically trained study physicians obtained a detailed medical history and current medication use. Participants were asked whether a physician had ever told them that they had the disease or health problem, and when the particular health condition had last been present (within the past four weeks; within the past 12 months; more than 12 months ago; don’t know). Current medication use was defined as any prescription or over-the-counter (OTC) drug used within 7 days prior to the survey interview. Participants were asked to bring the original medication containers or prescriptions to the survey centre for the purpose of verification. For each medication brand name, daily dosage and duration of use were documented and the ATC-(Anatomical Therapeutic Chemical) codes were assigned [37].

For the present analysis, we used information regarding the following 25 chronic health problems: hypertension, hyperlipidemia, hyperuricemia/gout, diabetes mellitus, stroke, myocardial infarction (MI), angina pectoris or other coronary heart disease (CHD), heart failure, asthma, chronic bronchitis, gastritis or duodenitis, gastric or duodenal ulcer, gall bladder disease, cirrhosis of the liver, thyroid disease, any malignant disease, any mental health problem (e.g. depression, anxiety disorder, psychosis), osteoarthritis, rheumatoid arthritis, osteoporosis, allergic rhinitis, allergic contact exzema, neurodermatitis, food allergy, urticaria. Etiologically or clinically related health conditions were further grouped into disease categories, such as cardiometabolic risk factors (hypertension, hyperlipidemia, hyperuricemia/gout); CVD (stroke, MI, angina pectoris or other CHD, heart failure); lower respiratory disease (asthma, chronic bronchitis); upper gastrointestinal tract disease (gastritis or duodenitis; gastric or duodenal ulcer); musculoskeletal disease (osteoarthritis, rheumatoid arthritis, osteoporosis), and atopic disease (allergic rhinitis, allergic contact exzema, neurodermatitis, food allergy, urticaria). Atopic disease did not consider allergic asthma, since differentiation between allergic and non-allergic asthma was not possible.

In order to assure capture of an ongoing health problem, the case definition of most of the considered health conditions required that the disease or health problem had ever been diagnosed by a doctor and that it had been present during the 12 months prior to the survey interview. Additional information on current drug use was considered for hypertension (ATC-Code C02, C03, C07, C08, C09), hyperlipidemia (ATC-Code C10), hyperuricemia (ATC-Code M04), diabetes (ATC-Code A10), and thyroid diseases (ATC-Code H03). Participants on relevant current medication were categorized as having the particular health problem irrespective of self-reported medical history. A lifetime medical history (ever diagnosed by a doctor) regardless of the time of last occurrence was used to define health conditions likely to involve irreversible or progressive organ damage (stroke, MI, angina pectoris or other CHD, any malignant disease).

### Covariates

Information on age, smoking habits (non-smoker, ex-smoker or current smoker), education, household income and professional status group was assessed using a self-administered standardized questionnaire. A composite socio-economic status index was computed integrating information on educational level, income and profession as previously described [38]. Scores from 1-7 were attributed to each of the components and the sum of the scores was calculated ranging from a possible minimum of 3 to a maximum of 21. Categories of social status were defined lower (3-8), intermediate (9-14), and upper (15-21).

### Statistical analysis

All statistical analyses were performed with SAS release 9.2 (SAS Institute, Cary, NC). Tests were two-sided, and P values <0.05 were considered statistically significant. A survey specific weighting factor took into account differences in demographic characteristics from the official German population according to age, gender, community size and residence in East or West Germany resulting from the sample design. The sampling weight ensured representative results from this sample for the total population in order to assure representativeness at the population level.

Statistical analyses were consistently stratified by gender. Continuous variables were presented as mean and 95% confidence intervals, categorical variables were expressed as a percentage of the population. Descriptive statistics were used to assess the crude prevalence in percent and 95% confidence intervals (CI) of persons with chronic health conditions by BMI and WC categories as defined above. In case of aggregate disease categories, associations were analyzed for disease groups as well as individual health conditions within groups. The independent association of overweight, obesity and abdominal obesity with comorbidities was analyzed in multivariate logistic regression models adjusting for covariates which included age as a continuous variable, and social status and smoking as categorical variables. Separate models were fitted for each health condition (dependent variable) and for overweight, obesity or abdominal obesity (independent variable). Odds ratios for the relationship between abdominal obesity and health conditions are adjusted for covariates as well as for BMI as a categorical variable. Interaction between overweight, obesity or abdominal obesity and age (< 50 vs. ≥ 50 years) was tested by adding the respective product terms to the multivariate models. Age specific estimates are presented where the interaction term was statistically significant.

## Results

**Figure 1:**
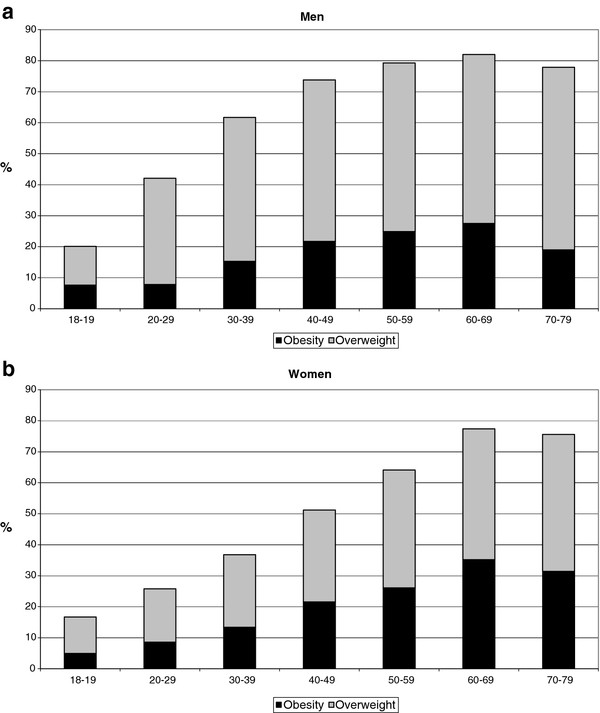
**Prevalence of men (1a) and women (1b) with overweight (BMI 25.0-29.9 kg/m**^**2**^**) and obesity (BMI ≥ 30 kg/m**^**2**^**) by age group.**

One third of men and less than half of women had normal weight based on BMI measurements. 48% of men fell into the overweight BMI category as compared to 31% of women. About 20% of men and women were obese with a BMI of more than 30 kg/m^2^ (Table 1). The prevalence of persons with overweight was consistently higher among men than among women of all age groups. In contrast, the prevalence of obesity in men and women was quite similar up to an age of 60 years, and was higher among women than men in older age groups (Figure 1).

**Table 1:** Baseline characteristics of the study population

	**Total**	**Men**	**Women**
N	7013	3417	3596
Age (years)	46.2 (45.5 – 46.8)	45.0 (44.3 – 45.8)	47.3 (46.6 – 48.0)
BMI (kg/m^2^)	26.6 (26.5 – 26.8)	26.9 (26.8 – 27.1)	26.3 (26.1 – 26.6)
Waist circumference (cm)	90.6 (90.1 – 91.1)	96.4 (95.9 – 96.9)	85.0 (84.4 – 85.6)
(%)			
Age groups (years)			
18-19	2.7	2.9	2.6
20-29	15.3	16.2	14.5
30-39	21.8	23.0	20.7
40-49	18.2	18.8	17.6
50-59	17.0	17.3	16.8
60-69	15.0	14.5	15.5
70-79	9.9	7.4	12.3
Body Mass Index (kg/m^2^)			
<25.0	40.3	33.2	47.2
25.0–29.9	39.4	48.0	31.2
30.0–34.9	15.4	15.7	15.1
≥35.0	4.9	3.1	6.5
Waist circumference (cm)			
<94/80	41.1	41.9	40.3
94-102/80-88	25.0	28.4	21.7
≥102/88	33.9	29.7	38.0
Social status			
Low	23.0	19.5	26.4
Middle	55.4	55.7	55.1
High	21.6	24.8	18.5
Smoking status			
Current	32.6	37.2	28.2
Ex	21.7	28.3	15.3
Never	45.6	34.5	56.4

**Figure 2:**
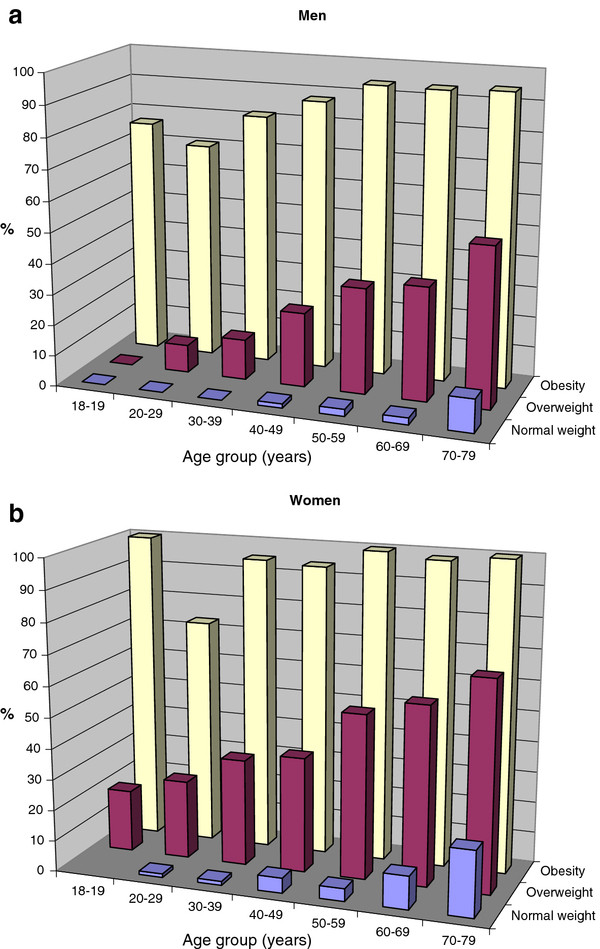
**Proportion of men (2a) and women (2b) with abdominal obesity (men ≥ 102 cm; women ≥ 88 cm) according to age group and body mass index categories (normal weight: BMI < 25.0 kg/m**^**2**^**; overweight: BMI 25.0-29.9 kg/m**^**2**^**; obesity: BMI ≥ 30.0 kg/m**^**2**^**).**

Overall 30% of men and 38% of women were classified as having abdominal obesity. In all age groups, the overall prevalence of abdominal obesity rose from 1.2% among normal weight men and 4.2% among normal weight women to 26.4% and 48.5% in overweight men and women, respectively, and to 88% and 96% among obese men and women (data in detail not shown). The prevalence of abdominal obesity was consistently higher in women than men in all age and BMI strata. In nearly all age groups, the majority of obese men and women had abdominal obesity (Figure 2).

The crude prevalence of persons with any cardiometabolic risk factor, diabetes, any CVD or osteoarthritis significantly increased with overweight and obesity in both sexes (Tables 2a and 2b). In women, this was also true for the prevalence of gall bladder disease. Overweight and obesity were not significantly associated with prevalent lower respiratory disease, upper gastrointestinal tract disease, liver cirrhosis, thyroid disease, cancer, mental health problem or musculoskeletal conditions other than osteoarthritis. Prevalent atopic disease was inversely related with overweight and obesity in both sexes, but a graded relationship across BMI categories was observed only in men.

**Table 2:** Prevalence (%, 95% CI) of self-reported physician-diagnosed morbidities according to BMI categories among German men (2a) and women (2b) 18-79 years of age

				
**a. Men (N = 3.417)**				
**BMI**	**Total**	**Normal weight <25 kg/m**^**2**^	**Overweight 25–29,9 kg/m**^**2**^	**Obesity ≥30 kg/m**^**2**^
N	3417	1131	1623	663
1. Cardiometabolic risk factor				
Hypertension	20.2 (18.6 – 21.9)	8.5 (6.9 – 10.5)	21.3 (19.1 – 23.6)	38.1 (34.0 – 42.4)
Hyperlipidamia	15.8 (14.4 – 17.4)	8.3 (6.6 – 10.3)	17.8 (15.8 – 20.1)	24.0 (20.3 – 28.2)
Hyperuricemia or gout	7.8 (6.9 – 8.8)	2.2 (1.5 – 3.3)	8.7 (7.2 – 10.5)	15.2 (12.5 – 18.3)
1 risk factor	31.1 (29.2 – 33.1)	15.0 (13.1 – 17.2)	34.1 (31.5 – 36.9)	52.0 (47.5 – 56.4)
2 risk factors	10.6 (9.6 – 11.8)	3.7 (2.7 – 5.0)	11.4 (9.7 – 13.3)	21.0 (17.9 – 24.4)
3 risk factors	2.1 (1.6 – 2.6)	0.4 (0.2 – 1.0)	2.3 (1.6 – 3.3)	4.3 (3.0 – 6.1)
2. Diabetes mellitus	4.2 (3.6 – 4.9)	2.1 (1.4 – 3.3)	4.8 (3.9 – 5.9)	6.4 (4.7 – 8.5)
3. Any CVD	8.8 (7.8 – 9.9)	4.2 (3.1 – 5.7)	10.2 (8.8 – 11.8)	13.4 (10.7 – 16.6)
Angina pectoris/other CHD	6.5 (5.7 – 7.5)	2.7 (1.9 – 3.9)	7.6 (6.3 – 9.1)	10.6 (8.2 – 13.6)
Myocardial infarction	3.3 (2.7 – 4.0)	0.9 (0.5 – 1.8)	3.7 (2.8 – 4.7)	6.3 (4.4 – 9.1)
Any CHD	7.3 (6.3 – 8.3)	3.1 (2.2 – 4.3)	8.3 (6.9 – 9.9)	12.1 (9.5 – 15.3)
Heart failure	2.4 (1.8 – 3.2)	1.0 (0.5 – 1.9)	2.9 (2.1 – 4.1)	3.7 (2.4 – 5.5)
Stroke	1.4 (1.1 – 1.9)	0.9 (0.4 – 1.7)	1.8 (1.3 – 2.6)	1.4 (0.7 – 2.9)
4. Any lower respiratory disease	7.5 (6.4 – 8.7)	7.1 (5.6 – 9.0)	7.1 (5.7 – 8.8)	9.1 (7.0 – 11.6)
Asthma	3.5 (2.9 – 4.3)	3.6 (2.5 – 5.1)	3.2 (2.4 – 4.3)	4.3 (2.9 – 6.3)
Chronic bronchitis	4.5 (3.7 – 5.5)	3.9 (2.9 – 5.4)	4.7 (3.6 – 6.1)	5.1 (3.7 – 7.0)
5. Any upper GIT disease	6.9 (6.0 – 7.8)	6.5 (5.0 – 8.3)	7.1 (5.9 – 8.6)	7.0 (5.3 – 9.4)
Gastritis/Duodenitis	6.6 (5.7 – 7.5)	6.0 (4.5 – 7.8)	6.8 (5.6 – 8.3)	6.8 (5.1 – 9.1)
Gastric/duodenal ulcer	1.3 (0.9 – 1.7)	1.5 (0.9– 2.4)	1.2 (0.8 – 1.9)	0.9 (0.4 – 2.1)
6. Gall bladder disease	5.1 (4.3 – 6.1)	3.4 (2.4 – 4.7)	5.5 (4.4 – 6.9)	7.3 (5.3 – 9.9)
7. Cirrhosis of the liver	0.2 (0.1 – 0.4)	0.1 (0.0 – 0.7)	0.2 (0.1 – 0.6)	0.5 (0.2 – 1.6)
8. Thyroid disease	6.9 (6.0 – 8.0)	5.5 (4.2 – 7.2)	7.8 (6.5 – 9.5)	7.1 (5.3 – 9.4)
9. Any malignancy (cancer)	3.0 (2.5 – 3.7)	3.1 (2.1 – 4.3)	3.2 (2.3 – 4.3)	2.6 (1.5 – 4.5)
10. Mental health problem	4.2 (3.5 – 4.9)	3.0 (2.1 – 4.3)	5.1 (4.1 – 6.4)	3.7 (2.4 – 5.5)
11. Any musculoskeletal disease	28.9 (26.9 – 31.0)	17.9 (15.4 – 20.7)	31.4 (28.5 – 34.4)	42.0 (37.3 – 46.8)
Osteoarthritis	27.7 (25.6 – 29.8)	17.1 (14.7 – 19.9)	30.1 (27.2 – 33.0)	40.3 (35.7 – 45.0)
Rheumatoid arthritis	2.9 (2.3 – 3.6)	2.3 (1.5 – 3.5)	2.8 (2.0 – 4.0)	4.0 (2.6 – 6.1)
Osteoporosis	0.7 (0.5 – 1.1)	0.6 (0.2 – 1.4)	0.7 (0.4 – 1.3)	0.9 (0.4 – 2.0)
12. Atopic disease*	25.0 (23.4 – 26.6)	30.7 (27.8 – 33.8)	23.1 (21.0 – 25.4)	19.6 (16.6 – 22.9)
**b. Women (N = 3.596)**				
**Body mass index (BMI)**	**Total**	**Normal weight <25 kg/m**^**2**^	**Overweight 25–29,9 kg/m**^**2**^	**Obesity ≥30 kg/m**^**2**^
N	3596	1690	1109	797
1. Cardiometabolic risk factor				
Hypertension	24.9 (23.2 – 26.7)	10.1 (8.5 – 12.0)	30.0 (27.1 – 33.0)	49.7 (46.1 – 53.3)
Hyperlipidamia	16.4 (14.9 – 18.0)	10.9 (8.9 – 13.2)	19.2 (16.4 – 22.3)	24.5 (20.9 – 28.6)
Hyperuricemia or gout	3.9 (3.2 – 4.8)	1.0 (0.6 – 1.6)	4.5 (3.3 – 6.3)	9.4 (7.1 – 12.3)
1 risk factor	33.7 (31.8 – 35.6)	18.2 (16.0 – 20.7)	40.1 (36.8 – 43.6)	58.0 (54.5 – 61.5)
2 risk factors	10.0 (8.8 – 11.3)	3.6 (2.6 – 5.0)	11.7 (9.5 – 14.4)	21.4 (17.7– 25.5)
3 risk factors	1.6 (1.2 – 2.1)	0.2 (0.1 – 0.6)	1.8 (1.0 – 3.2)	4.2 (2.8 – 6.4)
2. Diabetes mellitus	4.9 (4.1 – 5.9)	1.6 (1.0– 2.5)	6.1 (4.6 – 8.0)	10.5 (8.1 – 13.5)
3. Any cardiovascular disease (CVD)	8.8 (7.5 – 10.2)	4.1 (3.2 – 5.4)	11.6 (9.0 – 14.8)	14.7 (11.8 – 18.2)
Angina pectoris/other CHD	5.2 (4.3 – 6.4)	2.3 (1.5 – 3.4)	7.0 (5.0 – 9.6)	9.2 (7.4 – 11.5)
Myocardial infarction	1.7 (1.2 – 2.3)	0.5 (0.2 – 1.1)	2.8 (1.7 – 4.5)	2.7 (1.7 – 4.1)
Any CHD	5.6 (4.6 – 6.9)	2.3 (1.6 – 3.5)	7.7 (5.6 – 10.6)	9.8 (7.8 – 12.2)
Heart failure	3.4 (2.8 – 4.2)	1.6 (1.1 – 2.6)	4.2 (3.1 – 5.7)	6.1 (4.2 – 8.8)
Stroke	1.7 (1.2 – 2.3)	0.7 (0.3 – 1.4)	2.5 (1.5 – 4.2)	2.5 (1.5 – 4.2)
4. Any lower respiratory disease	7.5 (6.6 – 8.6)	7.5 (6.2 – 9.1)	6.6 (4.9 – 8.8)	8.8 (6.7 – 11.4)
Asthma	4.1 (3.4 – 5.0)	4.3 (3.3 – 5.5)	4.0 (2.7 – 5.9)	4.0 (2.8 – 5.8)
Chronic bronchitis	4.3 (3.6 – 5.2)	4.0 (3.0 – 5.2)	3.5 (2.4 – 5.2)	6.0 (4.3 – 8.3)
5. Any upper GIT disease	8.6 (7.5 – 9.8)	8.8 (7.3 – 10.6)	7.7 (6.1 – 9.7)	9.4 (7.3 – 12.1)
Gastritis/Duodenitis	8.3 (7.2 – 9.4)	8.5 (7.0 – 10.2)	7.4 (5.8 – 9.4)	9.0 (6.9- 11.6)
Gastric/duodenal ulcer	1.1 (0.8 – 1.6)	1.0 (0.6 – 1.8)	0.9 (0.4 – 1.8)	1.6 (0.9- 2.7)
6. Gall bladder disease	15.0 (13.6 – 16.4)	6.9 (5.6 – 8.4)	17.4 (15.1 – 20.0)	29.0 (25.3 – 33.0)
7. Cirrhosis of the liver	0.1 (0.0 – 0.2)	0.1 (0.0 – 0.5)	0.1 (0.0 – 0.5)	0.1 (0.0 – 0.8)
8. Thyroid disease	26.2 (24.4 – 28.1)	24.1 (21.8 – 26.5)	28.6 (25.8 – 31.6)	27.4 (24.1 – 31.0)
9. Any malignancy (Cancer)	4.7 (3.9 – 5.6)	3.8 (2.8 – 5.1)	5.8 (4.4 – 7.4)	5.1 (3.4 – 7.4)
10. Mental health problem	9.2 (8.2 – 10.5)	8.2 (6.6 – 10.3)	10.0 (8.2 – 12.1)	10.4 (8.2 – 13.0)
11. Any musculoskeletal disease	33.1 (30.9 – 35.4)	22.5 (20.0 – 25.3)	40.0 (36.6 – 43.5)	46.3 (41.8 – 50.8)
Osteoarthritis	29.0 (26.7 – 31.3)	18.5 (16.1 – 21.3)	35.3 (31.6 – 39.1)	42.6 (37.9 – 47.4)
Rheumatoid arthritis	5.3 (4.3 – 6.3)	3.8 (2.8 – 5.1)	6.3 (4.8 – 8.3)	6.9 (5.1 – 9.3)
Osteoporosis	5.9 (5.0 – 7.0)	4.6 (3.6 – 5.8)	6.9 (5.3 – 8.8)	7.6 (5.7 – 10.0)
12. Atopic disease*	39.2 (36.9 – 41.5)	43.1 (40.1 – 46.2)	35.3 (32.4 – 38.4)	36.3 (32.1 – 40.8)

*Allergic rhinitis, allergic contact exzema, neurodermatitis, food allergy or urticaria; *GIT* = gastrointestinal tract; *CHD* = coronary heart disease.

After adjustment for age, social status, and smoking, both overweight and obesity were significantly associated with cardiometabolic risk factors and osteoarthritis in men and women (Table 3). Compared to men with normal weight, overweight men had an 84% higher chance (OR = 1.84, 95% CI: 1.47; 2.31) and obese men had a fourfold higher chance (OR = 4.07, 95% CI; 3.16; 5.25) of also having any cardiometabolic risk factor (Table 3). Similarly, overweight men had a 1.4 higher odds (OR = 1.41 (1.12-1.78)) and obese men had a 2.1 higher odds (OR = 2.14 (1.65-2.76)) of having osteoarthritis than normal weight men. The strength of these associations was similar albeit slightly weaker among women. Sex-specific associations of overweight and obesity with cardiovascular risk factors, but not with osteoarthritis were stable across age strata, comparing persons below 50 years of age and those 50 years and older. Obesity was associated with a significant 1.8-fold higher odds for CVD among men and a 1.5-fold higher odds among women. Only among women, overweight and obesity were also positively associated with diabetes (OR = 1.85 (1.03-3.30) and OR = 2.94 (1.63-5.31)) as well as gall bladder disease (OR = 1.65 (1.22-2.25) and OR = 3.06 (2.26-4.14)). Atopic disease was inversely related to overweight in both sexes and with obesity in men. Results persisted in sensitivity analyses excluding underweight individuals with BMI less than 18.5 kg/m^2^.

**Table 3:** Association of self-reported physician-diagnosed morbidities and overweight, obesity and abdominal obesity among German men and women 18-79 years of age

					
**Men (N = 3320)**	**Normal weight BMI <25 kg/m**^**2**^	Overweight* BMI 25–29.9 kg/m^**2**^	Obesity* BMI ≥30 kg/m^**2**^	**No abdominal obesity WC <102 cm**	Abdominal obesity** WC ≥102 cm
**N**	1089	1584	647	2287	1033
Any cardiometabolic risk factor	1.0	**1.84 (1.47 - 2.31)**	**4.07 (3.16 - 5.25)**	1.0	1.23 (0.95 - 1.60)
Age <50 years	1.0	**2.19 (1.58 - 3.05)**	**4.28 (2.84 - 6.46)**		
Age ≥50 years	1.0	**1.51 (1.07 - 2.12)**	**3.58 (2.41 - 5.32)**		
Diabetes mellitus	1.0	1.35 (0.81 - 2.28)	1.69 (0.91 - 3.15)	1.0	1.56 (0.95 - 2.57)
Any cardiovascular disease (CVD)	1.0	1.33 (0.89 - 2.0)	**1.82 (1.13 - 2.93)**	1.0	1.10 (0.73 - 1.64)
Gall bladder disease	1.0	1.13 (0.75 - 1.69)	1.50 (0.91 - 2.45)	1.0	0.97 (0.62 - 1.53)
Osteoarthritis	1.0	**1.41 (1.12 - 1.78)**	**2.14 (1.65 - 2.76)**	1.0	1.13 (0.87 - 1.46)
Age <50 years	1.0	**1.57 (1.14 - 2.17)**	**2.35 (1.62 - 3.41)**		
Age ≥50 years	1.0	1.06 (0.80 - 1.40)	**1.53 (1.06 - 2.22)**		
Atopic disease	1.0	**0.79 (0.64 - 0.97)**	**0.67 (0.51 - 0.88)**	1.0	1.11 (0.84 – 1.48)
**Women (n = 3470)**	**BMI <25 kg/m**^**2**^	**BMI 25–29.9 kg/m**^**2**^	**BMI ≥30 kg/m**^**2**^	**WC <88 cm**	**WC ≥88 cm**
**N**	1647	1069	754	2158	1312
Any cardiometabolic risk factor	1.0	**1.48 (1.14 - 1.92)**	**3.40 (2.60 - 4.46)**	1.0	**1.44 (1.08 - 1.92)**
Age <50 years	1.0	1.20 (0.85 - 1.70)	**2.87 (2.02 - 4.09)**		
Age ≥50 years	1.0	**1.59 (1.14 - 2.22)**	**3.66 (2.52 - 5.31)**		
Diabetes mellitus	1.0	**1.85 (1.03 - 3.30)**	**2.94 (1.63 - 5.31)**	1.0	**3.18 (1.69- 5.99)**
Any cardiovascular disease (CVD)	1.0	1.34 (0.82 - 2.19)	1.51 (0.97 - 2.34)	1.0	0.93 (0.51 – 1.70)
Gall bladder Disease	1.0	**1.65 (1.22 - 2.25)**	**3.06 (2.26 - 4.14)**	1.0	**1.73 (1.25 - 2.39)**
Osteoarthritis	1.0	**1.38 (1.10 - 1.74)**	**1.65 (1.25 - 2.17)**	1.0	1.12 (0.86 - 1.46)
Age <50 years	1.0	**1.48 (1.06 - 2.08)**	1.45 (0.99 - 2.14)		
Age ≥50 years	1.0	1.24 (0.93 - 1.66)	**1.53 (1.10 - 2.12)**		
Atopic disease		0.91 (0.76 - 1.09)	1.14 (0.91 - 1.42)	1.0	**0.75 (0.59 – 0.95)**

*BMI* = Body Mass Index *WC* = Waist Circumference.

* adjusted for age (continuous), social status, (low, middle, high), smoking (ex, never, current).

** adjusted for age (continuous), social status (low, middle, high), smoking (ex, never, current), and categories of BMI (<25 kg/m^2^, 25- < 30 kg/m^2^, ≥30 kg/m^2^).

Among women but not among men, abdominal obesity, independent of covariates and BMI categories, was significantly and positively related to cardiometabolic risk factors (OR = 1.44 (1.08-1.92)), diabetes (OR = 3.18 (1.69-5.99)) and gall bladder disease (OR = 1.73 (1.25-2.39)), and significantly and inversely associated with atopic disease (OR = 0.75 (0.59-0.95)). No significant association of abdominal obesity with CVD or osteoarthritis was observed in either sex (Table 3).

## Discussion

This study presents estimates of the cross-sectional relationship between overweight and obesity and several chronic conditions and diseases in a representative sample of German adults. The prevalence of overweight and obesity increased with increasing age and the percentage of men and women with abdominal obesity rose steadily with age in overweight and even in the normal weight persons, as defined by BMI.

The prevalence of the majority of chronic conditions and diseases rose with increasing BMI. Both overweight and obesity in men and women were significantly associated with cardiometabolic risk factors and osteoarthritis, which is consistent with findings from the representative telephone survey (Behavioral Risk Factor Surveillance System, BRFSS) in the United States among adults aged 18 years and older [31]. This cross-sectional study showed a strong association between overweight and obesity, and cardiometabolic risk factors such as high blood pressure, high cholesterol, and arthritis. Several large cohort studies also found a direct positive association between high BMI and hypertension [6,39], and increased BMI and osteoarthritis [23]. A systematic review and meta-analysis of prospective cohort studies on comorbidity related to obesity and overweight demonstrated a significant association between overweight and obesity with incident diabetes, CVD, asthma, gallbladder disease, osteoarthritis, and various types of cancer [40]. Furthermore, our survey results indicated a positive association among men and women between overweight and obesity and diabetes and gallbladder disease, which is also consistent with findings from large cohort studies [8,9,19,20]. However, in our study, the relationship between overweight and obesity and diabetes and gallbladder disease was only significant among women. The stronger association between obesity and gallbladder disease has been observed in other studies, but the reason still remains unclear [41,42]. The missing significance between overweight and obesity and diabetes among men may be due to the lower prevalence of diagnosed diabetes in men compared to women. The diabetes diagnosis in our study based on a self-reported physician diagnosis and the current drug use without any blood glucose analyses. In Germany, the prevalence of undiagnosed diabetes is higher among men than among women [43,44]. Therefore, the true association between overweight and obesity and diabetes in men could be underestimated.

The increased risk for CVD among overweight and obese has been investigated in several prospective studies. In the Framingham Heart Study, a prospective cohort study with 44 years of follow-up, both men and women with overweight had a two-fold higher, obese men had a 1.46 times higher (95%-CI: 1.20-1.77) and obese women a 1.64 times (1.37-1.98) higher risk for CVD [7]. A meta-analysis of cohort studies including more than 300.000 persons indicated a 30% higher risk for CHD in overweight and an 80% higher risk in obese men and women [14]. However, in our study the association between overweight and CVD were neither significant in men nor in women, and for obesity and CVD we only observed a significant higher risk in men. The missing significance among women could be due to gender disparities in the diagnosis of the underlying diseases in the CVD group, in particular CHD. In fact, analyses by subgroups confirmed that the observed sex difference is related to CHD. More than a decade ago, when this survey was conducted, the diagnosis of CHD was a typical male diagnosis and not very commonly diagnosed among women [45]. Heart failure is a weak diagnosis compared to other disease endpoints, and should be interpreted with caution. The diagnosis stroke is probably under diagnosed in a national health survey, as participants with serious diseases and disabilities would not have participated.

Regarding obesity and allergic diseases the published literature is controversial and the reasons for inconsistent study results remain unclear. Two non-representative cross-sectional studies found an independent effect of overweight on atopic diseases only among women, but not among men. The Humboldt Study 2003 collected data from around 2000 18-79 year old Canadians [26] and Kilpeläinen et al. 2006 included 10667 Finish students aged 18-25 years [46]. In contrast, representative data from the NHANES Survey 2005-2006 indicate that obesity was not independently associated with atopy [30]. Nevertheless, our study indicated a significant inverse association between abdominal obesity and atopic disease in women and overweight and obesity and atopy in men. The differences between study findings might be due to different underlying diagnosis criteria. In the Humboldt Study 2003 allergic diseases were assessed by a questionnaire and skin prick test [26] and NHANES used a positive immune essay test result to define atopy [30]. The cross-sectional study with young Finns used a physician diagnosis of allergic rhinitis or allergic conjunctivitis and in our study participants were asked whether a physician had ever told them that they had an atopic disease (e.g. allergic rhinitis, allergic contact exzema, neurodermatitis, food allergy, urticaria). More research is needed to clarify the comparability of different diagnosis information.

As in the descriptive analyses of prevalence estimates across BMI categories, we did not observe an independent significant association of overweight and obesity with lower respiratory disease, upper GIT disease, and cirrhosis of the liver, thyroid disease, malignancy or mental health problem in the multivariate analysis.

In fact, after adjustment for BMI, abdominal obesity was associated with higher odds ratios for cardiometabolic risk factors, diabetes mellitus and gall bladder diseases, but the estimates did not show significance among men. This might be due to the use of WHO recommendations for the sex specific cut-offs for high risk WC (men: ≥102 cm; women: ≥88 cm) [36]. Although results from a longitudinal cohort study showed that even WC values below these cut-offs are associated with increased risk of diabetes in men and women, it could be that the thresholds for men might be inappropriately set to detect significant associations between WC and cardiometabolic risk factors and diabetes mellitus [47]. Furthermore, while men had larger mean values for WC compared to women the percentage of men with abdominal obesity is lower than among women. It seems that the used WC cut-off is a better predictor among women compared to men.

To our knowledge, the relationship between weight status and prevalence of different conditions and chronic diseases has not been described for the German population. These presented results are an important baseline investigation for further longitudinal analyses of upcoming representative data from the next survey. This survey includes participants of GNHIES and, therefore, will allow us to compare the obesity associated disease burden between two surveys at different time points (1997-1999 and 2008-2011) of the last decade.

Our study has several limitations. First, the presented data on comorbidity of overweight and obesity is restricted to the population 18 to 79 years. Thus, our results cannot be generalized to persons 80 years of age and older. Second, given the cross-sectional design, the results of the present study do not permit any conclusions on causality or causal directions between obesity and comorbidities. Third, the use of self-reports to identify chronic diseases can lead to recall and misclassification bias. Self-reports have been widely used in epidemiological studies to assess the burden of chronic diseases. For most conditions specificity of self-reported information is high, in particular if additional information on medication use is used for internal validation [48]. In the present study, we considered medication use for hypertension, hyperlipidemia, hyperuricemia, diabetes, and thyroid disease. We cannot exclude, however, that we missed participants with undiagnosed or subclinical disease. For example, we only considered a physician-diagnosed “mental health problem” and did not use additional information based on the World Mental Health Composite Diagnostic Interview administered in a subset of the study population [49]. We also did not consider the available blood pressure or serum lipid measurements, because objective measurements to define subclinical or previously undiagnosed disease were available for only some conditions. Finally, it is possible that selection and survivor bias contributed to underestimate the burden of comorbidity associated with overweight and obesity. In particular, men and women with serious conditions likely to cause irreversible organ damage, such as stroke or malignancy would be expected to be under-represented in this national survey.

In addition, BMI is a fairly blunt instrument through which to define overweight and obesity and does not adequately reflect body composition, especially unfavorable fat mass distribution. BMI does not discriminate between body fat mass and lean tissue mass and it is possible that we have underestimated the abdominal fatness in older individuals, who have higher percentages of fat in relation to lean muscle mass compared to younger men and women. Therefore, we analyzed WC as a marker for abdominal fat mass.

Despite these limitations the results of the present study are based on a large nationally representative sample of 18-79 year old adults in Germany and a selection bias can be neglected. Our cross-sectional results on comorbidity of overweight and obesity are in line with large prospective studies focusing on single health outcomes, and the above mentioned biases might not affect our results substantially.

## Conclusion

This study provides nationally representative data on comorbidity of overweight and obesity in Germany. Current classification systems of obesity focus only on anthropometric measures and should be complemented by information on presence or extent of comorbidities, e.g. Edmonton obesity staging system [50]. Obesity as a major cause of morbidity is a substantial public health problem with economic impact [51]. Co-occurring chronic health problems affect more than half of the elderly population [52], and will thereby led to an increasing burden of disease associated with overweight and obesity.

## Competing interests

The authors declare that they have no conflicts of interest.

## Authors’ contributions

AS, GBMM and CSN designed the study. AS conducted the statistical analysis. AS, GBMM and CSN interpreted the data and contributed to the writing of the manuscript. GM was involved in the design and conduction of GNHIES. All authors read and approved the final manuscript.

## Pre-publication history

The pre-publication history for this paper can be accessed here:

http://www.biomedcentral.com/1471-2458/12/658/prepub
